# Antiproliferative effect of low-level laser/ photobiomodulation on gingival fibroblasts derived from calcium channel blocker-induced gingival overgrowth

**DOI:** 10.1007/s10103-024-04122-y

**Published:** 2024-07-25

**Authors:** Melis özgül slezovic, Işıl Saygun, Vahdi Umut Bengi, Muhittin Serdar, Alpdogan Kantarci

**Affiliations:** 1 Department of Periodontology, University of Health Sciences, Gulhane Faculty of Dental Medicine, Ankara, Turkey; 2 Department of Medical Biochemistry, Acıbadem Mehmet Ali Aydınlar University, Ankara, Turkey; 3 Forsyth Institute, Cambridge, Massachusetts, USA

**Keywords:** Gingival enlargement, Calcium channel blockers, Low level laser therapy, Photobiomodulation, CTGF, TGF- β, Collagen

## Abstract

The aim of this study was to evaluate the antiproliferative properties of low-level laser therapy (LLLT) on gingival fibroblasts obtained from calcium channel blocker-induced gingival overgrowth (GO). Gingival fibroblasts of patients with GO were compared to healthy gingival fibroblasts (H). Both cells were exposed to LLLT (685 nm wavelength, 25mW power, diode laser) and compared to those not treated with LLLT. Cell proliferation and viability were measured with MTT assay at baseline and after 24 and 72 h. TGF-β1, CTGF, and collagen Type 1 levels were evaluated with Enzyme-Linked Immunosorbent Assay (ELISA). LLLT significantly decreased the proliferation of GO fibroblasts (*p* < 0.05) while leading to a significantly higher proliferation in H fibroblasts compared to the untreated cells (*p* < 0.05). GO cells showed significantly higher CTGF, TGF-β, and collagen Type 1 expression than the H cells (*p* < 0.05). LLLT significantly reduced CTGF levels in GO cells compared to the control group (*p* < 0.05). In H cells, CTGF and TGF-β levels were also significantly decreased in response to LLLT compared to the control group (*p* < 0.05). While LLLT significantly reduced collagen expression in the H group (*p* < 0.05), it did not significantly impact the GO cells. LLLT significantly reduced the synthesis of the growth factors and collagen in both groups with an antiproliferative effect on the gingival fibroblasts from calcium channel blocker-induced GO, suggesting that it can offer a therapeutic approach in the clinical management of drug-induced GO, reversing the fibrotic changes.

## Introduction

Gingival overgrowth (GO) develops due to inflammation, drug side effects, and genetic and neoplastic processes [1]. Anticonvulsant, immunosuppressive, and calcium channel blockers (CCB) lead to drug-induced GO [2]. Calcium channel blocker-induced GO is characterized by cell proliferation, extracellular matrix (ECM) deposition in the gingival connective tissue with varying degrees of inflammation and fibrosis and disruption of collagen metabolism [3] regulated by Transforming Growth Factor-β (TGF-β) [4]. TGF-β controls collagen levels through cellular communication network (CCN) proteins, a family of ECM-related proteins involved in intercellular signaling. Connective tissue growth factor (CTGF), also known as CCN2, is a member of the CCN family; TGF-β1 rapidly and strongly induces CTGF and contributes to regulating the ECM in GO [5]. CTGF is an important factor in collagen production by regulating the proliferation and differentiation of fibroblasts and stimulating tumor growth and metastasis [6].

Drug-induced GO has a high prevalence, and the fact that many factors such as inflammation, drug dose, agent of the drug used, host response, and genetic predisposition play a role in the pathogenesis makes treatment difficult. Since it is not always possible to discontinue or change the drugs that cause GO, developing treatments to prevent recurrence is critical. The necessity for the development of novel, non-invasive therapeutic approaches becomes apparent.

Low-level laser therapy (LLLT) has been used in many fields of medicine as a non-invasive and non-thermal method for its anti-fibrotic and photobiomodulation effects [7]. While initially expressions such as ‘photobioactivation’ and ‘biostimulation’ were frequently used to describe the stimulation effect of low-level lasers, the term ‘photobiomodulation’ began to be used after the inhibitory effect was observed [8]. The effects of low-level laser therapy (LLLT), also known as photobiomodulation therapy (PBM), in alleviating the negative consequences of inflammation and fibrosis by inducing repair in tissues, have been demonstrated in numerous studies [9, 10]. LLLT or PBM is emerging as a promising new treatment option for fibrosis in different organs. However, the anti-fibrotic potential of this treatment in dentistry needs to be elucidated and the cellular and molecular interactions of the laser need to be clarified. Various studies in dentistry have shown that low-level laser therapy (LLLT) and photobiomodulation (PBM) can accelerate cell proliferation, improve wound healing, and be suitable for treating different oral diseases [11, 12]. However, it is unknown whether LLLT affects GO gingival fibroblasts differently than cells from healthy (non-GO) individuals. Therefore, we tested the hypothesis that LLLT would reduce cell proliferation of GO-derived fibroblasts and affect the levels of TGF-β, CTGF, and collagen, which are involved in fibrotic processes.

## Materials and methods

This study was ethically approved by the Health Sciences University Scientific Research Ethics Committee with decision number 2021 − 371 and supported by the Health Sciences University Scientific Research Projects Unit with project number 2022/003.

### Primary cell culture from GO individuals

After obtaining informed consent from a patient with calcium channel blocker-induced GO in need of surgical excision, the excised gingival tissues were transferred to the laboratory by placing the excised gingival tissues in a 5 mm Petri dish containing 14 mm Dulbecco Modified Eagle Medium (DMEM) with 1100 U/ml penicillin and 100 µg/ml streptomycin. The gingival tissues were cut into small pieces and transferred into a 25-flask with a sterile syringe tip. 10% medium (10% fetal calf serum- 1% penicillin − 1% L-glutamine) was added and placed in an oven containing 5% CO2 at 37 °C for 72 h. The medium was refreshed every 72 h until all 80% confluency. After trypsinization, the cells were transferred to a large 75 flask. Cells between passages 4 and 7 were used at 3×${10}^{4}$ cells/well in 24-well plates. Human gingival fibroblasts (HGF-1- ATCC CRL-2014; American Type Culture Collection, Manassas, VA) were used as a healthy control (H). Healthy gingival fibroblasts were incubated in DMEM (Sigma D6429 containing 4500 mg/l glucose, sodium pyruvate, and sodium bicarbonate) medium supplemented with 10% heat-inactivated fetal calf serum FBS (Biochrom S0115) and 100 U/ml penicillin, 100 µg/ml streptomycin (Biochrom A2213) and incubated at 37 °C in a 95% humidified oven with 5% CO2. These cells were seeded 3×${10}^{4}$ cells/well in 24-well plates.

### Low-level laser treatment

**Fig. 1:**
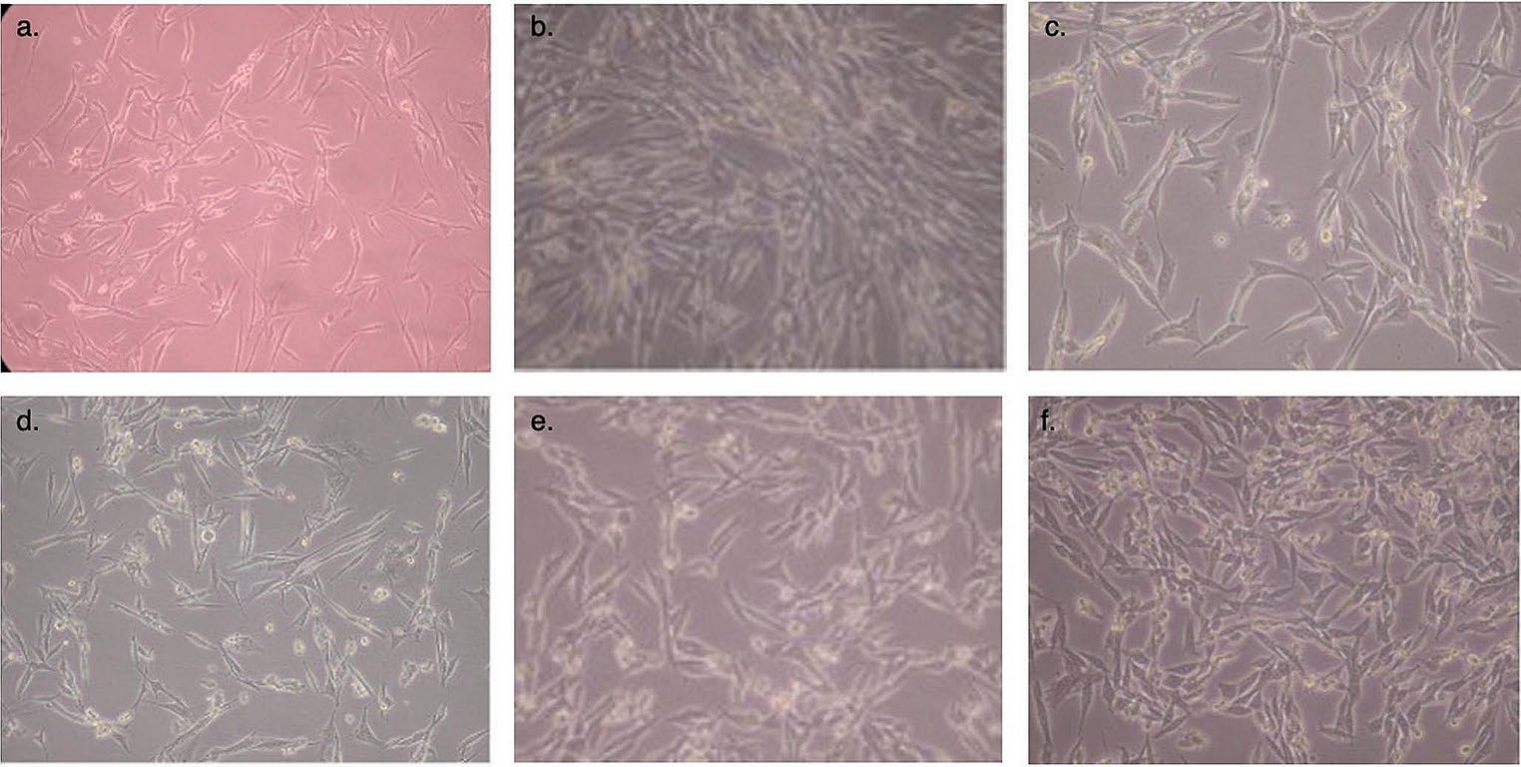
**a.** Initial GO cells (x 4 magnification) **b.** Untreated control GO cells ( x10 magnification) **c**. GO cells after LLLT ( x10 magnification) **d.** Initial H cells ( x10 magnification) **e.** Untreated control H cells ( x 10 magnification) **f.** H cells after LLLT ( x10 magnification)

Each group was divided into LLLT and non-treated control groups. Irradiation was performed in continuous mode with a diode laser (BTL-2000 Benešov Czech Republic) at a wavelength of 685 nm, a power output of 25 mW and with a power density of 2.0 J/${cm}^{2}$ for 100 s. The laser beam was transmitted by an optical fiber and irradiated to a circular area of 1 ${\text{c}\text{m}}^{2}$. Laser irradiation was applied in two doses. The second dose was repeated 3 days after the first dose. Laser application was not applied to the control groups (Fig. 1).

### Cell viability and proliferation

MTT (3-(4,5-dimethylthiazol-2-yl)-2,5-diphenyltetrazolium bromide) (Sigma, St Louis, MO, USA) was used to evaluate the cytotoxicity and cell proliferation of the experimental groups at baseline and after 24 h. Briefly, fibroblasts were seeded in 96-well plates. The media were replaced with fresh media, and MTT (5 mg/mL) solution was added into the wells in one-tenth of the original culture volume, and the incubation was continued at 37 °C in 5% CO_2_ for 4 h. Then, the incubation medium was removed, and 100 µL 0.04 N isopropanol was added to each well. The plate was incubated at room temperature for approximately 30 min. The absorbance was measured at 570 nm in a microplate reader (Biotek Epoch, Germany).

### Enzyme-linked immunosorbent assays (ELISA)

Supernatants were frozen at -80 °C until the analysis. TGF-β1, CTGF, and collagen type I (Col-I) levels were measured using ELISA kits (Fine Test Human TGF-β1 ELISA Kit, sensitivity level 18.75 pg/ml, Wuhan, China; Fine Test Human CTGF ELISA Kit, sensitivity level 37.5 pg/ml, Wuhan, China; Fine Test Human Collagen Type I (Col I) ELISA Kit, sensitivity level 0.821 µg/ml, Wuhan, China).

Statistical Analysis Statistical analyses were performed using SPSS (SPSS Inc. Chicago, IL, USA). Shapiro-Wilks test was used to evaluate whether the data were normally distributed. The significance of the difference between the means of the groups in MTT was assessed by the Kruskal-Wallis test. Bonferroni test was used for pairwise post-hoc comparisons. Analysis of Variance (ANOVA) was used to determine whether the growth factor release levels of the study groups differed from each other. Tukey-Kramer test was used to determine which groups differed. Differences below *p* < 0.05 were considered significant.

## Results

### Effect of LLLT on the proliferation of gingival fibroblasts

**Fig. 2:**
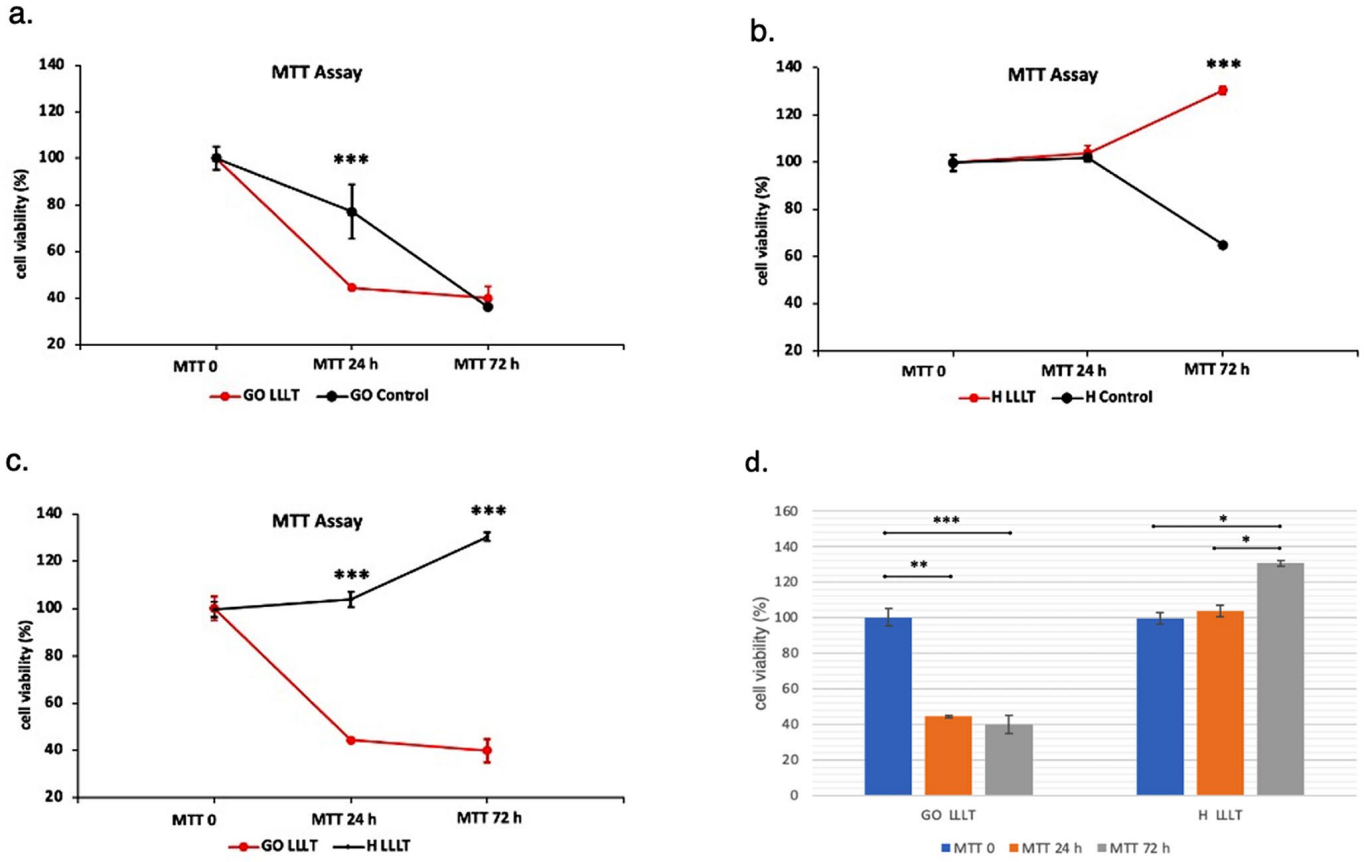
**a.** Comparison of MTT levels between GO cells **b.** Comparison of MTT levels between H cells **c.** Comparison of MTT levels between laser-treated GO and H cells. **d.** Intra-group MTT comparison by time points. MTT 0: Baseline MTT, MTT 24: 24th-hour MTT, MTT 72: 72nd-hour MTT. MTT 0: Baseline MTT, MTT 24: 24th hour MTT, MTT 72: 72nd hour MTT *: *p* < 0,05 **: *p* < 0,01 ***: *p* < 0,001

The proliferation of GO laser-treated cells was statistically lower than that of control GO cells after the first dose. In contrast, no significant difference was detected between the GO cells after the second dose (Fig. 2a). While a significant difference was seen in the 24th and 72nd hours GO cells in response to LLLT compared to baseline, there was no significant difference between these time points (Figs. 1 and 2d). While there was no significant difference between the baseline and 24 h in H cells in response to LLLT, there was an increase in H cell proliferation after 72 h (Fig. 2b, d). While there was no statistical difference between the baseline viability and proliferation of GO and H cells, LLLT-treated GO cells showed significantly lower proliferation after 24 and 72 h compared to the H group (Fig. 2c).

### Effects of LLLT on TGF-β, CTGF and collagen expressions

**Fig. 3:**
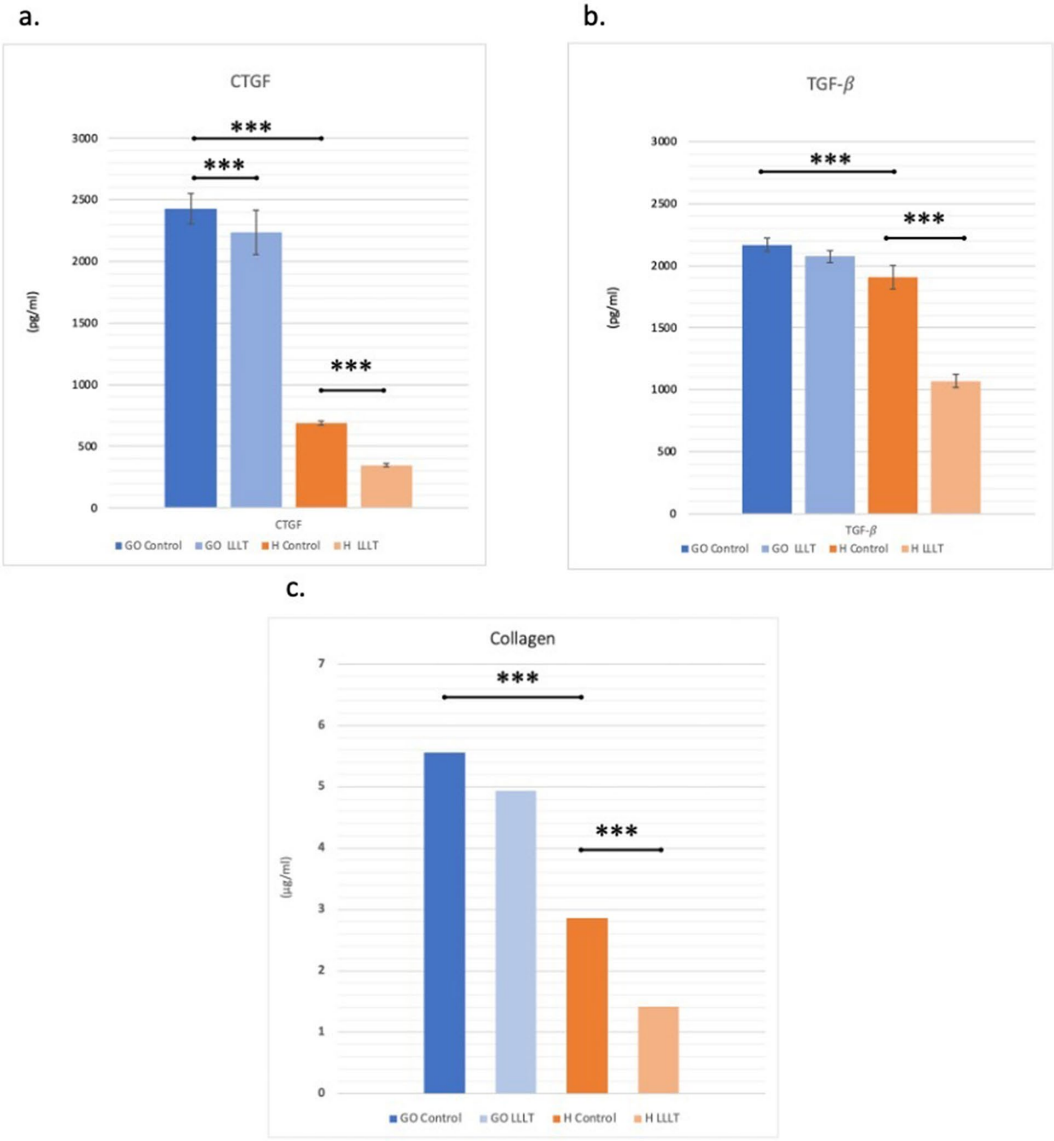
**a**. Comparison of CTGF levels between groups **b.** Comparison of TGF- β levels between groups. **c.** Comparison of collagen levels between groups. *: *p* < 0,05 **: *p* < 0,01 ***: *p* < 0,001

Untreated control GO cells showed significantly higher CTGF levels than the control H group. LLLT significantly reduced CTGF levels in both GO and H cells (Fig. 3a). Untreated control GO cells showed significantly higher TGF-β1 levels than control H cells. The reduction of TGF-β1 levels in GO cells in response to LLLT was not statistically significant. LLLT resulted in a significant decrease in TGF-β1 levels in H cells (Fig. 3b). Untreated control GO cells showed significantly higher collagen levels than control H cells. LLLT was found to reduce collagen levels in both GO and H cells. However, this effect was only statistically significant in H cells (Fig. 3c). A high positive correlation was found between TGF-β1 and CTGF (*r* = 0.798; *p* < 0.0001). A low positive correlation was found between CTGF and collagen (*r* = 0.335 *p* < 0.05).

## Discussion

LLLT suppressed proliferation in cells obtained from CCB-induced GO without showing a cytotoxic effect, while it stimulated proliferation in healthy cells. A similar proliferative effect of LLLT in healthy cells has also been shown in previous studies [13, 14]. In a previous in vitro study, we divided human gingival fibroblast cells into 3 groups. In the first group, the cells were irradiated with a single dose of diode laser, the second group was irradiated with the same dose of laser for two consecutive days, and the third group, which was not irradiated with diode laser, served as the control group. Both irradiated groups showed higher proliferation and viability than the control group [15]. In another in vitro study on healthy gingival fibroblasts obtained from a cell bank, gingival fibroblasts were divided into experimental and control groups. The experimental group received LLLT with a diode laser for 3 consecutive days, while the control group received no treatment. A significantly higher cell proliferation was detected in the experimental group at 48 and 72 h [16]. In contrast, some studies did not report cell proliferation [17], possibly due to differences in the characteristics of the laser device, such as dose wavelength, irradiation mode, and the characteristics of the cell type. It is crucial to employ adequate dosimetry in LLLT. A fundamental principle, termed the “biphasic dose response,” has emerged, indicating that larger doses of light tend to be less efficacious than smaller doses [18]. However, no consensus has been reached on the optimal range. In studies, a diode laser with a wavelength ranging from 660 to 940 nm was frequently used in continuous mode, with a large range in output power ranging from 10 to 500 mW, while the energy density range ranged from 1 to 15.8 J/cm^2^ [19]. In our study, LLLT were performed in 2 doses, similar to the parameters of our previous studies in which the photobiomodulation effect of LLLT was demonstrated [15, 20].

LLLT has been reported to be beneficial in reducing fibrosis in various organs [7, 21]. Still, there is no study on the anti-fibrotic effect of LLLT in drug-induced gingival growths. In our study, while proliferation was suppressed with the first dose of LLLT in cells obtained from CCB-induced gingival growth, no significant difference in cell proliferation value was observed after the second dose of LLLT. However, a decrease in the proliferation level of the control group without LLLT was observed at 72 h. In primary cell cultures, cells have a limited lifespan and proliferation capacity decreases over time [22]. The increase in the number of these cells, which are thought to have high proliferation potential, in the wells may have caused the environmental conditions to be insufficient for cell proliferation and maintenance of cell viability. In contrast, cells of the same character treated with LLLT simultaneously showed a stable level of viability, a remarkable result of the biomodulation property of the low-level laser.

In the pathogenesis of gingival overgrowth, extracellular matrix (ECM) accumulation and fibrotic changes occur by releasing excessive amounts of mediators that contribute to regulating fibrogenic and regenerative signals with increased fibroblasts [23]. In our study, TGF-β levels were significantly higher in cells obtained from CCB-induced growths than in healthy cells, consistent with previous studies [24, 25]. A decrease in TGF-β levels was observed in these hyperplasic cells and healthy fibroblasts treated with LLLT compared to non-laser-treated cells. Studies in which the anti-fibrotic effect of LLLT was demonstrated were generally performed in the medical field [26, 27]. No studies in the literature evaluated the anti-fibrotic properties of LLLT on gingival growths and its effect on the expression of growth factors thought to play a role in fibrosis. TGF- β levels of healthy gingival fibroblasts treated with LLLT were shown to be significantly lower than those of untreated cells [11]. The researchers stated that TGF-β is a versatile growth factor and may play a role in undesirable wound healing models such as fibrosis and scar tissue formation. In the study by Kuo et al. on keloid tissues obtained from patients, the effect of LLLT on gene expression was evaluated, and it was reported that LLLT mediated the blockade of TGF- β expression [28]. It is thought that TGF-β can slow down the degradation of connective tissue matrix by decreasing the synthesis of matrix metalloproteinases and plasminogen activators [29].

Recent studies have shown that TGF-β controls CTGF expression may have effects on subsequent steps of tissue fibrosis [30]. CTGF is involved in several important biological functions, including cell proliferation, differentiation, adhesion, and angiogenesis, and in controlling multiple pathological processes such as tumor development and tissue fibrosis [31]. The literature shows increased CTGF levels are generally demonstrated in drug-induced growths and patients with hereditary gingival fibromatosis [32, 33]. Our study detected significantly high CTGF levels in cells obtained from CCB-induced growths. These cells showed very high CTGF release even when their proliferation decreased, CTGF is a reliable biomarker in drug-induced gingival overgrowth. In our study, the increased CTGF release detected in the cells obtained from CCB-induced gingival overgrowth was suppressed by LLLT, and the CTGF level in LLLT-treated cells was found to be significantly lower in healthy gingival fibroblasts compared to the control group.

Suppression of excessive synthesis of CTGF in healthy cells, which is a biomarker for drug-induced gingival overgrowth, may provide a prophylactic benefit in terms of the occurrence of gingival hyperplasia. Similar to the results of our study, Yeh et al. showed in vitro that LLLT inhibited the transcriptional activity of CTGF in cells obtained from patients with oral submucous fibrosis [34]. In another study supporting these results, Zhu et al. reported that CTGF mRNA expression decreased with laser irradiation in gingival fibroblasts obtained from keloid tissues [35].

Our study detected increased TGF- β, CTGF and collagen levels in cells obtained from CCB-induced growths. Similarly, Fuji et al. showed increased collagen levels in their study with gingival fibroblasts obtained from patients using nifedipine [36]. Lu et al. reported that fibroblast cultures formed from gingival tissues obtained from patients with nifedipine-induced gingival overgrowth exhibited increased expression of type I collagen compared to fibroblasts obtained from healthy gingiva [37]. Similarly, there are other studies in the literature showing that collagen, TGF-β1 and CTGF levels increase in drug-induced gingival enlargement and hereditary gingival fibromatosis [38, 39]. Pisoschi et al. reported in their study that the TGF-β-CTGF relationship was activated in gingival growth caused by calcium channel blockers [40]. Another study reported that TGF- β acts as a strong stimulatory signal for connective tissue formation in fibrotic conditions and acts together with CTGF in its growth-stimulating effect [41]. Some authors have reported that the relationship between CTGF and TGF-β may be a mediator that strengthens the fibrogenic function of fibroblasts [42, 43]. In our study, a positive correlation was observed between TGF-β and CTGF and between collagen and CTGF, similar to previous studies.

## Conclusion

This is the first study to demonstrate that LLLT has a modulating effect on cell proliferation in gingival fibroblasts from tissues with gingival hyperplasia due to the use of calcium channel blockers without causing cytotoxicity and can suppress the synthesis of CTGF, TGF-β, and collagen—all of which have been linked to the pathogenesis of gingival hyperplasia. Low-dose laser applications suppress the proliferation of fibroblasts isolated from hyperplasic tissue while stimulating the proliferation of healthy cells. This indicates that LLLT can be used in growth therapies without causing any pathological effects in the mouth.

In conclusion, for LLLT to be included in clinical practice as a new treatment model in treating hyperplasia in dentistry, in vitro studies evaluating biological markers for pathogenesis and randomized controlled clinical trials evaluating long-term recurrence are needed.
